# Implementation of an Electronic Medication Management System in a large tertiary hospital: a case of qualitative inquiry

**DOI:** 10.1186/s12911-021-01584-w

**Published:** 2021-07-27

**Authors:** Milan Rasikbhai Vaghasiya, Jonathan Penm, Kevin K. Y. Kuan, Naren Gunja, Yiren Liu, Eui Dong Kim, Neysa Petrina, Simon Poon

**Affiliations:** 1 School of Computer Science , The University of Sydney, Camperdown, NSW 2006 Australia; 2 Western Sydney Local Health District, Westmead, NSW 2145 Australia; 3 Faculty of Medicine and Health, The University of Sydney, Camperdown, NSW 2006 Australia; 4 Faculty of Medicine and Health, School of Pharmacy, The University of Sydney, Camperdown, NSW 2006 Australia; 5 Department of Pharmacy, Prince of Wales Hospital, Randwick, NSW 2031 Australia

**Keywords:** Electronic health records, Implementation science, Electronic prescribing, Medical informatics, Technology assessment

## Abstract

**Background:**

Hospitals across Australia are implementing Clinical Information Systems, e.g. Electronic Medication Management Systems (EMMS) at a rapid pace to moderate health services. The benefits of the EMMS depend on the acceptance of the system by the clinicians. The study hospital used a unique patient-centric implementation strategy that was based on the guiding principle of “one patient, one chart” to avoid a patient being on a hybrid medication chart. This paper aims to study the factors facilitating or hindering the adoption of the EMMS as viewed by clinicians and the implementation team.

**Methods:**

Four focus groups (FG), one each for (1) doctors, (2) nurses, (3) pharmacists, and (4) implementation team, were conducted. A guide for the FG was based on the Unified Theory of Acceptance and Use of Technology (UTAUT).

**Results:**

A total of 23 unique subthemes were identified and were grouped into five main themes (1) implementation strategy, (2) organisational outcome of EMMS, (3) individual impact of EMMS, (4) IT product, and (5) organisational culture. Clinicians reported improvement in their workflow efficiency post-EMMS implementation. They also reported some challenges in using the EMMS that centered around the area of infrastructure, technical and design issues. Additionally, the implementation team highlighted two crucial factors influencing the success of EMMS implementation, namely: (1) the patient-centric implementation strategy, and (2) the organisation readiness.

**Conclusion:**

Overall, this study outlines the implementation process of the EMMS in a large healthcare facility from the clinicians’ and the implementation team’s perspectives using UTAUT model. The result suggests that clinicians’ acceptance of the EMMS was highly influenced by the unique implementation strategy (namely, patient-centric approach and clinical leadership in the implementation team). Whereas the level of adoption of EMMS by clinicians was determined by their level of perceived and realised benefits. On the other hand, a number of barriers to the adoption of EMMS were discovered, namely, general training instead of customised training based on local needs, technical and design issues and lack of availability of computer systems. It is suggested that promptly resolving these issues can improve the adoption of the EMMS.

**Supplementary Information:**

The online version contains supplementary material available at 10.1186/s12911-021-01584-w.

## Background

There is a growing interest in the use of technology in the health sector in Australia. Recently, the Australian government framed National Digital Health Strategy in 2017 [1]. The strategy acknowledges digital health as a priority for the Australian health care system and outlines seven strategic areas for improving health outcomes and service delivery. Some of the strategic areas include providing greater access to healthcare across Australia, supporting the efficient health system, and digitally-enabled models of care and centralising patient information [1]. As a result, hospitals across Australia are implementing Clinical Information Systems (CIS), e.g. Electronic Medication Management Systems (EMMS) at a rapid pace to moderate health services.

Various benefits of EMMS have been reported in previous studies, such as improving organisational efficiency, reducing medication errors, and minimising the cost of medication management processes [2–9]. Furthermore, studies suggested that EMMS improved the efficiency of medication management processes by reducing the time between prescription and administration of antibiotics to the patients [10, 11].

The benefits of EMMS are much dependent on how well clinicians embrace the new system. Clinicians are the key stakeholders that influence the success of the transition from the paper system to an electronic system. Earlier research found that nurses and physicians perceived EMMS beneficial as it improved their workflow and efficiency [12, 13]. On the contrary, other studies suggest that EMMS can lead to the introduction of a new type of errors, such as wrong medication selection [14, 15], increased workload among the clinicians [16], loss of productivity [17], loss of autonomy [18] and a clinician burnout [17, 19]. Previous studies have reported several human factors that can affect the adoption of the system including clinician’s perceived ease of use and usefulness of the system [20], clinician’s knowledge of the system [21], confidence in using the system [21], user involvement [22] and socio-technical aspect of the system design [23].

Furthermore, the role of the implementation team is critical during the implementation of EMMS [24]. While most research studies focus on understanding the clinicians’ perspective in using the system, little research has focused on understanding the implementation team’s perspective [25]. Implementation team forms the implementation strategy and oversees the process of implementing the new system [26]. Thus their experiences can provide valuable insight into understanding the implementation process.

## Patient-centric implementation strategy

Development of a comprehensive implementation strategy plays a vital role in the success of EMMS implementation [24]. By and large, healthcare organisations use two prominent methods of EMMS implementation: (1) Big Bang approach and (2) phased (staged) approach [17, 27, 28]. Both of these methods have their challenges. The big bang approach requires rapid change, increased system testing and a large support team to help the users during the implementation process [27]. On the other hand, the phased approach creates hybrid medication charts (paper and electronic charts), workflow interruptions and prolong the time to implement the system [27].

Keeping in mind the limitation of these two implementation approaches, the implementation team, consisted out of clinicians from various discipline, introduced “patient-centric implementation” strategy [27]. The strategy was based on the guiding principle of “one patient, one chart”. Each new patient started on the EMMS from day one of the implementation, and existing patients stayed on paper charts [27]. This patient-centric approach avoided hybrid medication charts being used for the same patient, thus minimising the risk of medication errors and also creating no disruption on the workflow.

## Conceptual framework

Various theories have been used in the literature to explain the users’ intention to use and actual use of the IT system in healthcare. The theory of reasoned action (TRA) and the theory of planned behaviour (TPB) has been used to understand behaviour intention and actual behaviour [29, 30]. TPB is an extension of TRA where individuals do not have complete control over their behaviour [30]. According to TPB, human behaviour is guided by three beliefs: (1) behavioural belief (user’s attitude) about the likely outcome of the behaviour, (2) normative belief (subjective norm) about the normative expectation of others and, (3) control belief (perceived behaviour control) about the internal and external factors that can facilitate or impede the performance of the users [30]. User’s Attitude, Subjective Norm and perceived Behaviour Control [30] in using the new system can influence the acceptance of the new system. In a big organisation, many external factors are beyond user’s absolute control (e.g. selection of the system, patient-focused implementation strategy, executive support, standards of practice, organisational culture, the training, the support provided to users and so forth) and this might influence user’s Behaviour Control.

**Fig. 1:**
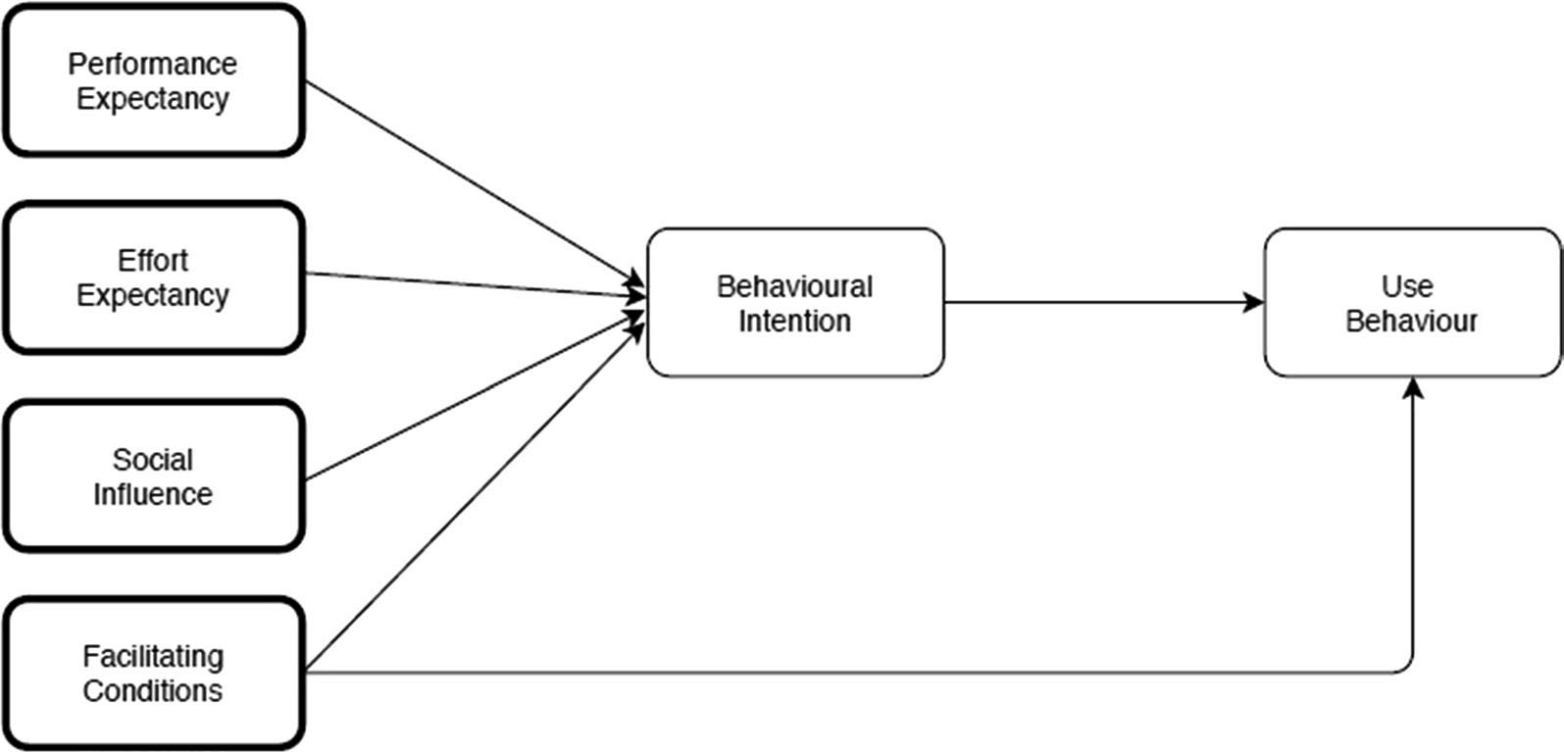
Unified theory of technology acceptance and use of technology [29]

Based on TRA, TPB, and six other theoretical models, Venkatesh [29] suggested the Unified Theory of Acceptance and Use of Technology (UTAUT) to explain technology acceptance. UTAUT [29] was used in this study to understand clinicians’ attitude in the adoption of the EMMS. UTAUT helps to explain how performance expectancy and effort expectancy (behavioural belief), social influence (normative belief) and facilitating conditions (control belief) affect behaviour intention and user behaviour (Fig. 1). We adopted the four core constructs of the UTAUT model, namely: (1) performance expectancy, (2) effort expectancy, (3) social influence, and (4) facilitating conditions [29] to create a protocol for the focus groups (FG) (Additional file 1: Appendix 1).

Therefore, this research aims to study the factors facilitating or hindering the adoption of the EMMS from clinicians’ and the implementation team’s perspective on patient-centric implementation strategy using the UTAUT framework.

## Methods

### Setting

The EMMS was implemented in one of the major tertiary teaching hospitals in Sydney, Australia. The study hospital is part of the local health district, which serves the population of more than two million people. The hospital had a capacity of 480 acute inpatient beds with an annual of 50,000 ED presentations.

The study hospital was the lead site for the Electronic Medical Record (EMR) implementation in New South Wales. It was also the first site to have full EMR utilisation before the EMMS implementation. The EMMS was implemented on 28th February 2017 in all clinical areas except for the intensive care unit (ICU) [27].

### Participants

Focus groups were held in the first few weeks of the implementation. The maximum variation sampling method was used to recruit participants from various clinical areas across the hospital. Twenty-nine staff from various clinical areas, including Emergency Department (ED), aged care, surgery, general medicine, cardiology, respiratory and cancer units, were recruited. Participants consisted of various age groups and with various levels of work experience. All participants were given a coffee voucher worth AU$ 5 as an appreciation for their participation. The composition of the FG was homogenous. All FGs had a mix of junior and senior clinicians and were from different age groups. Clinicians who were using EMMS were invited from all specialties except the intensive care unit (ICU). Clinicians in ICU used different medication system hence were excluded from the study. Students and assistants to clinicians were excluded from the study as well due to their limited scope of practice in using EMMS. The maximum variation sampling method was used to recruit participants from various clinical areas across the hospital. Clinicians who filled our prior survey with intent to participate were invited for participation in FG via email. We then approached different clinical units directly to recruit clinicians. Head of Pharmacy and Head of Implementation team were approached to see if they can assist in organising the FG. A total number of pharmacists working in the hospital were much smaller than doctors and nurses. We recruited all 13 pharmacists in FG to ensure we have sufficient representation from such clinician category. One participant dropped out in each nurses’ and doctors’ FG due to their busy workload.

### Focus group (FG) and questions

FGs were organised for each group of doctors, nurses, pharmacists and the implementation team. The FG guide was created based on the UTAUT [29] framework (see Additional file 1). Semi-structured questions were asked to participants using this guide. The FG was started with a question related to the specific construct and their responses were explored in detail by prompting a discussion. The discussion was kept fluid and each participant were encouraged by the researchers to contribute. The average duration of the FG was 60 min.

FGs were facilitated by both an academic (SP) and a Ph.D. student (MV). The academic has more than 20 years of experience in the evaluation of health information technology. The academic was assisted by a Ph.D. student in note keeping and recording of the FG. The Ph.D. student worked as a registered nurse for over 10 years in the acute facility and with 5 years of research experience. We felt that it was essential to have a team of academic and a clinician who can complement each other with their respective skills and avoid the bias and possibility of reflexivity in the data collection process.

FGs were organised according to staff preference and availability. Participants’ written and oral consents were obtained before the FG. The objective of the study, researchers’ role in the study and participants’ right to withdraw from the FGs were explained to participants at the start of the FG. Staff were introduced to the researchers and made aware of the recording of the FG for the research purpose while assuring the confidentiality of their views.

### Data processing and analysis

**Fig. 2:**
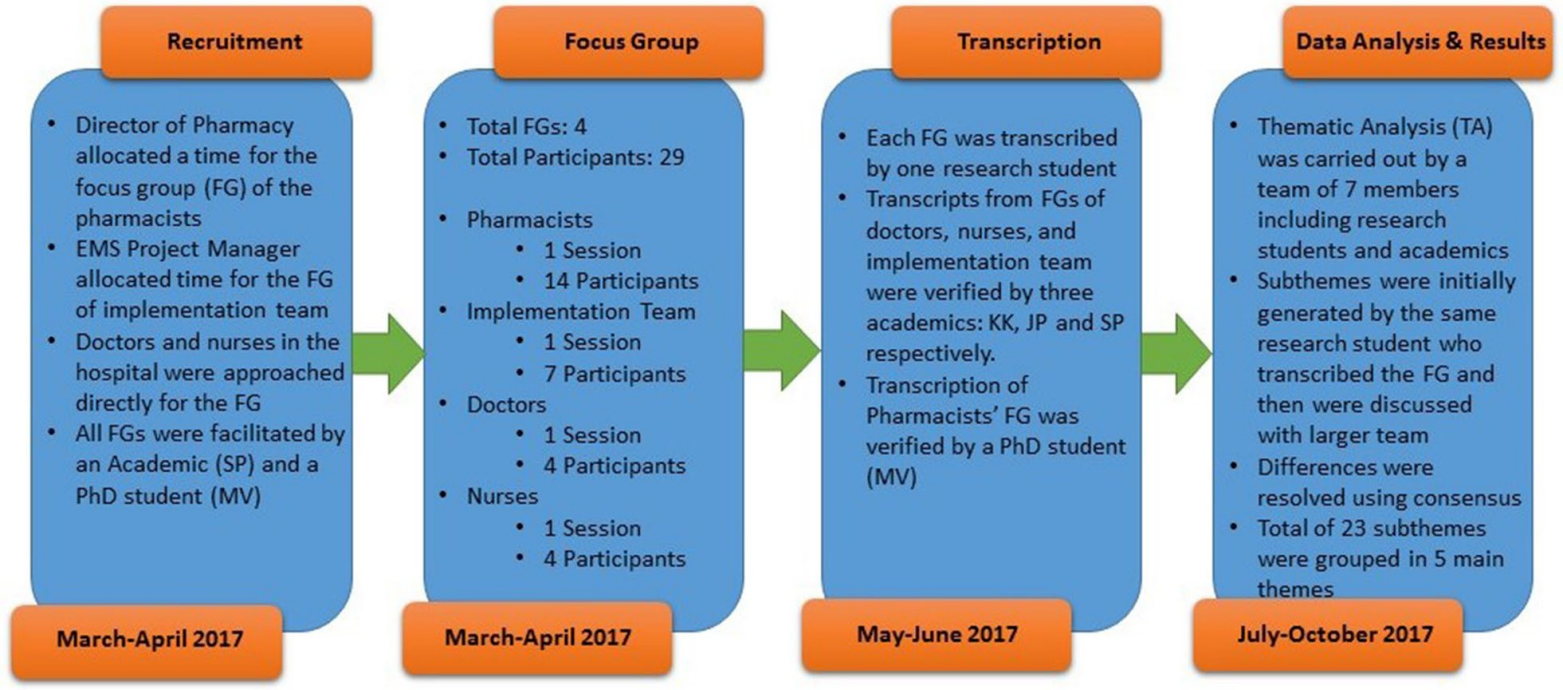
Research process

All FG recordings were stored on a secured computer. FGs were transcribed by two PhD students and two research students. Each transcript was fully reviewed by one academic staff to validate the text as well as to identify and remove any discrepancies. The research process is outlined in Fig. 2.

Thematic analysis (TA) of the transcript was performed using NVivo 11 (QSR International Pty Ltd released 2015, Version 1.0.1.1. TA is widely used in qualitative research. TA is a method to analyse qualitative data by extracting identifiable themes and subthemes [31]. During the coding process, each transcript was coded by two independent members of the research team. Once the individual members completed the coding, the group of seven members met to discuss discrepancies until unanimous agreement was reached for the subthemes and themes through open discussion. Subthemes that did not meet unanimous agreement were recategorised. If they could not be recategorised, they were dropped. In total, three rounds of meetings occurred. All themes and subthemes either reached a unanimous agreement or were recategorised and reached unanimous agreement.

Triangulation between different methods of data collection (e.g. survey and focus group) was not done as this manuscript only reports results from the qualitative focus groups. In regards to subthemes been highlighted between all user groups, our analysis shows some were unique to a specific group. For example, the subtheme of “administrative support” was more prominent in Implementation Team’s FG.

## Results

Twenty-three unique subthemes were identified and were grouped into five main themes: (1) implementation strategy, (2) organisational outcome of EMMS, (3) individual impact of EMMS, (4) IT product, (5) organisation culture (Table 1). More detailed descriptions of the themes, subthemes and quotes are included in Additional file 2.

**Table 1:** Themes, subthemes and quotes

Theme	Subtheme	Example quotes
Implementation strategy	Support during the implementation	“I find the helpline really useful as well. I have called them like five times, I think. They are really helpful.” (P7)
	Training	“More practice (with the system) and hands-on (training) is needed.” (D4)
	User engagement/ownership/involvement	“They (Clinicians) were heavily involved in that build process. There was an endocrinologist to put his hands up to help. He was heavily involved, and some of the nurses were involved as well. They accepted the system even prior to our go-live.” (IT1)
	Administrative support	“I think the big thing is that we had that governance that supported us. We had that leadership from the executive level from the beginning.” (IT1)
	Infrastructure	“It has helped a lot, improved what you said that, you know, with giving the medication, the duration, how many days to wean off (medications), its good in a way, I think it’s just a lack of the equipment.” (N2)
	Super-users	“Each ward has got a super-user.” (N1)
	Communication among the support staff	“That instant messaging, can’t speak enough [about it]. Not that I’ve used that much, but that’s what lots of other people did (during the roll-out).” (IT2)
	Capacity building	“The super user team would ensure that the receiving staff were able to do what they need to do.” (IT4)
Organisational outcome of EMMS	Legibility and information completeness	“The EMMS is useful because the medication orders are a lot clearer and we can read them easily.” (N2)
	Alerts and Prompts	“Emergency Department has made a folder of the most commonly used medications list (in EMMS), and you can choose from there. Each department is trying to make its own list to save time while ordering medications.” (D2)
	Access to the system	“I know it is quite useful to be anywhere in the hospital, I mean if you are in ED and if you would have to come all the way up to level six or seven, it is a big deal. So, it really helps in that sense too.” (D2)
	Visibility of information	“It (EMMS)changed the particular way that previously we look at a medication chart I would look at which dose from the pharmacokinetic and pharmacological point of view, which patient is considered lower priority which is the higher priority. So I do the high priority (task).” (P2)
	Workflow	“It changed the particular way that previously we look at a medication chart enabled to assess the clinical context.” (P2)
Individual impact of EMMS	Change in the way of working	“Before I would leave that job. Now do that job straight away, because it’s very easy thing to change.” (D4)
	Accountability	“That is right, you know whom to contact if there is an issue with a dose whereas before when you ring and say “I never charted that”, but (now) you can read on the top and see who charted it.” (P3)
	Self-efficacy	“There’s a lot of things each and every one of us has figured out. Like I figured out some things I’m dying to tell it to XX.” (P4)
IT product	Design and Build process (Process Design)	“So we looked at what the other sites had done and made it (the system) better basically.” (IT7)
	Design issues (System Design)	“That’s the only thing that’s a bit confusing if you do not read the full order sentences you would not know if it is paracetamol or paracetamol with codeine.” (N2)
	Technical issues	“Our other barrier is mainly to do with listings of product that do not match with what we have got and having to do all these unnecessary steps of having to change the products.” (P3)
	Workarounds	“I think I just like re-charted it like in a slightly different way. It’s a bit time-consuming.” (D4)
Organisation culture	Organisational readiness	“I think the facility has the right culture, and this is only something this hospital just brings itself to make changes to be innovative, develop and work around the new system and develop strategies.” (IT1)
	Communication with colleagues	“The interactions with other colleagues like doctors or pharmacist are better than before” (N1)
	Cultural factors	“Perhaps we could have some IT person to teach some of our different generation of practitioner how to use the short cut key instead of grabbing the mouse and waste a lot of time.” (P2)

### Mapping the results to the UTAUT Model

We mapped the themes and subthemes identified from the FG to UTUAT model, as shown in Table 2.

**Table 2:**
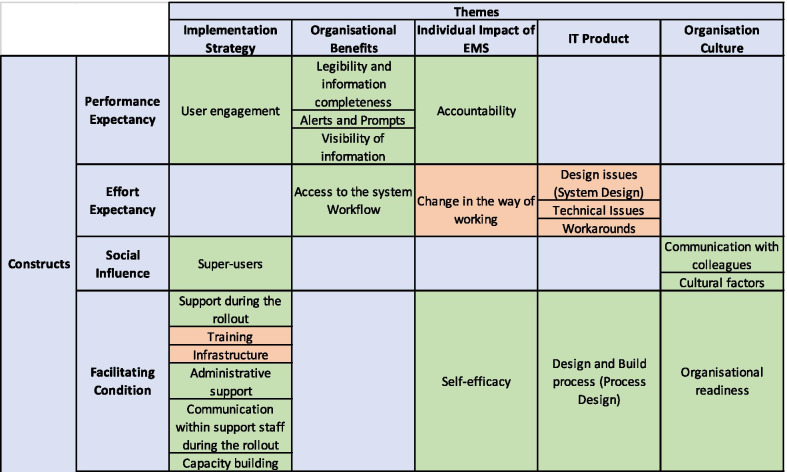
Themes and subthemes identified from the FG data mapped to constructs from Venkatesh’s [29] UTAUT model [29]

We identified potential facilitators (green) and barriers (orange) in the adoption of EMMS (Table 2). The majority of our subthemes fell under the construct of Facilitating conditions. Majority of these subthemes that were identified as a facilitator in the construct of Facilitating Conditions came from the theme of Implementation Strategy. The theme of IT product had no subthemes in the constructs of Performance expectancy and Social influence. The theme of Organisational benefits had no subthemes in the constructs of Social influence and Facilitating condition. Similarly, the theme of Organisation culture had no subthemes in the constructs of Performance expectancy and Effort expectancy.

### Implementation strategy

The study hospital used a patient-centric strategy to implement the EMMS. During the roll-out of EMMS, the implementation team focused on providing support at the entry points. Meanwhile, trained and motivated clinicians were given the title of “super-users” and assisted to support their colleagues within their respective wards.

The implementation team highlighted the importance of a patient-centric strategy to avoid hybrid medication charts as patients move from one location to other in the hospital.

There was a lot of discussion within the executive level, and our plan was to go patient-centric. We needed a certain amount of support staff for coverage (in the hospital), which we didn’t have. So we just put all the support staff at the entry point, like the ED, irrespective of day and time. And then supporting the wards by fairly extensive super user group that can pick up the work at the end level. (IT4).

I know in other sites, due to rapid roll-out (strategy), there were issues when transferring patients from one ward to another, because they were on a separate systems (paper vs EMMS). With a patient-centric approach, if you transfer a patient from one location to another, then you do not have to go back to paper and back to EMMS. (IT6).

From the clinicians’ FGs, overall, we received positive feedback about the implementation strategy, e.g. the support provided during the implementation, the training and user engagement.

- Support during the implementation

Support was available in each unit round the clock in the first two weeks of the EMMS implementation. A small team of support staff mainly focused on the arrival points, e.g. ED, Operation theatres, etc. The superuser group was responsible for providing support to the clinicians in the wards. Apart from the support staff and supper users, the dedicated helpline phone number was made available for the clinical staff if they face any difficulty with the system. Participants perceived the availability of support staff and helpline was useful.

I find the helpline really useful as well. I have called them five times. They are really helpful. (P7).

- Training

The training was provided to all clinicians in two parts. At go-live stage, 79% of doctors, 68% of nurses or midwives, and 90% of pharmacists were trained in the EMMS system [27]. The training was provided in two 2-h sessions before go live. The training was general in nature and clinicians were trained on how to perform the medication tasks specific to their role in the EMMS. Although participants found the training was useful and adequate, they suggested more specific scenario-based training and simulation-based training as it would help them in using the EMMS more effectively.

I would suggest that some stimulation training sessions on how to use the system before using the real system would be helpful. (P2).

- User engagement/ownership/involvement

User engagement in adopting the new system is important in the implementation of the system. The implementation team highlighted that a strong clinical lead made the stakeholder engagement easy due to their established relationship with their respective disciplines.

They (Clinicians) were heavily involved in that build process. There was an endocrinologist to put his hands up to help. He was heavily involved, and some of the nurses were involved as well. They accepted the system even prior to our go-live. (IT1).

The implementation team also highlighted the crucial role that the administrative support from the top of the organisation played in assisting the process of system implementation.

I think the big thing is that we had that governance that supported us. We had that leadership from the executive level from the beginning. (IT1)

- Infrastructure

Participants felt that the hospital infrastructure was not adequate to facilitate the completion of their daily tasks using EMMS. Nurses were concerned as there were not enough Workstations on Wheel (WOW) in high activity areas such as the Emergency Department.

The Work Stations on Wheels (WOW) are not always available for us to wheel around, it is just time-consuming sometimes, just because of not enough computers available. (N2)

### The organisational outcome of EMMS

This theme outlines the benefits of EMMS perceived by the participants. The perceived benefits that improved user acceptance and reduced their resistance to using EMMS include improved legibility, information completeness, alerts and prompts, access to the system from anywhere and improved visibility of medication information.

- Legibility & information completeness

Participants perceived the legibility and information completeness as a key benefit from the new system as it would reduce the chance of medication errors.

The EMMS is useful because the medication orders are a lot clear, and we can read them easily. (N2)

- Alerts and prompts

Participants felt that various features of the EMMS, e.g. alerts, prompts, and the ability to create a folder with the most commonly charted medications, were helping them to do their task more efficiently as clinicians don’t have to look for the most commonly used medication orders every time and can select them from pre-saved lists in the EMMS.

Emergency Department has made a folder of the most commonly used medications list (in EMMS), and you can choose from there. Each department is trying to make its own list to save time while ordering medications. (D2).

Participants also mentioned that the auto-population of the medication information was helping them in their tasks by saving time in re-charting the medications as they don’t have to manually write all the medication details and they can rather choose the medication orders from auto-populated dropdown list.

During the after-hour shifts, we have to re-chart a lot of meds that is saving us five to ten minutes per chart. I have found my after-hours shifts much, much more manageable as a result. (D4).

- Access to the system

The EMMS system was accessible from anywhere in the hospital. Participants mentioned that the new system saves time as they do not need to go to the different departments of the hospital physically.

I think it does help. I know it is quite useful to be anywhere in the hospital, I mean if you are in ED and you would have to come all the way up to level six or seven, it is a big deal. So, it really helps in that sense. (D2).

- Visibility of medication information

The new system improved the visibility of the medication information for clinicians. Pharmacy staff felt that they could prioritise their task based on their needs as they have better visibility of the information due to the new system.

### Individual impact of EMMS

Participants felt that the system has made everyone more accountable for their tasks as now there is a trail of the person who has performed specific tasks.

That is right, you know whom to contact if there is an issue with a dose whereas before when you ring and say “I never charted that”, but (now) you can read on the top and see who charted it. (P3).

On the contrary, pharmacists mentioned the verification of the medication order was challenging in the new system as they had to verify all the medication orders and it would appear outstanding in the system until they verify the medication orders. This functionality of EMMS created safer medication practice and added accountability, however, it contributed to extra workload.

Because of our expanded role in the computerised verification of all the medication orders, for those pharmacists who actually have to go to see the patient and take a history, it becomes the time factor. (P2).

### IT product

This theme outlines the subthemes related to the design and the build process (Process Design), design issues (System Design), and technical issues in the EMMS.

The implementation team outlined the benefits of having the content ready from the previously implemented EMMS in other sites.

So we looked at what the other sites had done and made it (the system) better basically. (IT7)

Clinicians mentioned some issues they faced with the design of the system e.g. inability to see the full medication order in the dropdown list and inability to print multiple medication scripts in one page. Nurses mentioned the possibility of errors when prescribing or reviewing the medications with similar names.

That’s the only thing that’s a bit confusing, if you do not read the full medication order sentence you would not know if it is paracetamol or paracetamol with codeine. (N2)

Apart from the design issues, participants also faced some technical issues with the EMMS. Pharmacist highlighted the issue of the medications that were not listed on their catalogue.

Our other barrier is mainly to do with listings of products that do not match with what we have got and having to do all these unnecessary steps of having to change the products. (P3).

Pharmacy staff also mentioned that the view of the medication lists was hindering their efficiency as the list of active medication was mixed with the cancelled and discontinued medications as well as some of the functionality in the system were American practice-based and were not aligned to the local practices.

The system basically is based on the American system, so the functionality is not made for the Australian pharmacists. So many functions inside (the system) are not necessary. It just creates confusion and then duplication. (P14).

### Organisation culture

The implementation team mentioned the organisational readiness of the hospital being instrumental in the EMMS implementation.

I think the facility has the right culture, and this is only something this hospital just brings itself to make changes to be innovative, develop and work around the new system and develop strategies. (IT1).

The EMMS also had an influence on the way clinicians interact with each other. Nurses mentioned that they do not see the pharmacists a lot in the ward post-EMMS implementation, but they felt that the job was still getting done.

## Discussion

This study outlines several factors that can facilitate the success of EMR adoption in a large hospital setting. First, the implementation strategy played an essential role as a facilitator in clinicians’ acceptance of the EMMS. The patient-centric implementation strategy adopted in this study is in line with clinicians’ core focus of patient safety. The well-thought-out approach of “one patient—one chart” gave ample time for clinicians to learn and adapt to the new system while avoiding the use of hybrid charts. This minimises the possibility of workflow interruptions and hence maximised users’ Behaviour Control compared to the more commonly used Big Bang or Phased (staged) approach.

Second, the support provided during the implementation was a critical factor in clinicians’ acceptance of the new system. Although, Information System implementation literature explains the relationship of the support in improving user acceptance of the EMMS [21], the type of support provided is also important. In the study hospital, the implementation team provided comprehensive support (e.g., the availability of the support staff 24/7, dedicated phone line and availability of technical staff to resolve any technical issues promptly) during the implementation of the EMMS. These various efforts taken by the implementation team improved the participants’ perceived belief of being successful in using the EMMS similar to Ajzen’s TPB—a person would be more likely to execute the behaviour if he/she is likely to be successful [30].

Third, the presence of clinicians in the implementation team further facilitated user acceptance in using the EMMS. The involvement of clinicians in implementation played a critical role in engaging with users as well as hospital executives to bring them on board during the change process. Clinicians felt comfortable to engaging with the implementation team as they felt easy to engage with them. This clinical leadership within the implementation team may have influenced the clinicians’ Attitude and Social influence in favor of adopting the EMMS.

Fourth, the benefits of EMMS highlighted by the participants are multifold, e.g. access to the system from anywhere, prompts and alerts, legibility and information completeness of medication orders and availability of all required information in one place. This perceived usefulness and perceived ease of use helped users to form a positive attitude towards using the system.

Fifth, this study found that organisation culture plays an essential role in the implementation of the EMMS. The study hospital took various initiatives in digital health domain previously and thus had the culture of adopting new technology. Positive organisation culture towards embracing the new system may have acted as a social influence in facilitating user’s behaviour intention to use the system as mentioned by Venkatesh et al. [26].

Participants also reported negative perceptions in using the EMMS. Doctors, nurses and pharmacists outlined the issue of not having enough computer units in some clinical areas as one of the main barriers in attending  to their routine tasks. Infrastructure is critical during the implementation of a new system [8], and inadequate infrastructure can pose as a barrier to the adoption of the EMMS.

Furthermore, clinicians also suggested scenario-based training instead of general training to prepare them better in using the EMMS in their respective clinical areas. Studies in the past have highlighted the importance of training [5, 15, 21, 23], but more tailored training according to the local needs can prepare clinicians in adapting to the new system quickly.

A significant number of technical and system design factors also influence the rate of user adoption. Although the literature suggests that some technical issues that arise during the system implementation do get resolved and the perception of the users in using the new system do change over time [32], these issues can further contribute to the new type of medication errors if they are neglected [15].

Furthermore, some of the issues raised by the clinicians (e.g. filling mandatory fields in the EMMS) were due to the change process of the system. This perceived loss of autonomy [18] with the new system contributes to the users feeling frustrated. Although clinicians may not like it, it was helping the organisation to monitor the medication process and generate detailed reports to see the improvements in the processes. Earlier research found similar results where the managers were benefiting from the new system, but the users had to do extra tasks in the new system [26]. The optimisation of the system to streamline the processes as well as conveying the benefits of completing these extra tasks can help the clinicians to perform the task efficiently [26] and improve the adoption of EMMS.

It was also interesting to see that three themes did not belong to at least two constructs of UTAUT model [29]. For example, the theme of IT product and Organisational culture had no subthemes related to the construct of Performance expectancy. One way to influence clinicians in adopting the system is to assess them how well they use the system in their daily tasks. We noticed that there was no such assessment of the clinicians in how well they use the EMMS. Assessing the clinicians on how well they use the system or having assessment criteria of using the EMMS as a part of clinicians’ performance review can also improve the adoption of the EMMS.

Findings of this study, including the unique implementation strategy, comprehensive support during the roll-out, clinicians being part of the implementation team, organisation culture, are crucial to the success of CIS and can be used in healthcare institutes globally.

### Limitations

There were four FGs organised for each group, e.g. nurses, doctors, pharmacists and the implementation team. While we had a good representation of the participants in the pharmacists’ and the implementation team’s FGs, we could not involve more participants in nurses’ and doctors’ FGs. Therefore we could not reach data saturation for these two clinical groups. Limited participation of nurses and doctors from various specialties was attributed to their busy workload during the implementation. It is possible that having more participants in nurses’ and doctors’ FG may uncover additional themes.

During the pharmacy FG, we were only able to meet with all the pharmacists at once and too many participants in the FG led to some participants not being able to provide their views and more vocal participants dominating the FG.

## Conclusion

Overall, this study provides significant insight in explaining the implementation process of EMMS in a large healthcare facility using UTAUT model. The unique implementation strategy with the patient-centric approach and clinical leadership in the implementation team played a crucial role in clinicians having a positive attitude towards EMMS. One of the facilitating condition, namely, comprehensive support provided by the implementation team, was influential in the adoption of EMMS. Perceived and realised benefits, e.g. clarity in medication orders, access to the system from anywhere and information completeness helped clinicians forming a positive attitude in the adoption of EMMS. On the other hand, general training instead of customised training based on local needs, technical and design issues and lack of availability of computers can act as a barrier to the adoption of the system. Promptly resolving these issues can give volitional control [30] to the clinicians and can assist in the success of the adoption.

Finally, our research adds a significant piece of knowledge to Health and Information Technology literature that the implementation strategy can influence all three domains (Attitude, Subjective Norm, Behaviour Control) of Ajzen’s TRA and TPB [30] and can contribute hugely to the successful adoption of EMMS. Healthcare institutes across Australia and beyond can use these results to better understand the factors affecting CIS implementation.

## Future research

Our finding of Implementation Strategy strongly associated with Attitude, Subjective Norm, Behaviour Control based on Ajzen’s TRA and TPB is based on a qualitative research method (FG data). Future research can combine a mixed method study with a correlational study to produce richer results in improving the utilisation of Health Information Technologies within healthcare settings.

## Supplementary Information

**Additional file 1.** Focus group guide. Questions that were used with during FGs.

**Additional file 2.** Themes, Subthemes and Quotes. Material collated from the analysis of FG data.

## Data Availability

The datasets generated and/or analysed during the current study are available from the corresponding author on reasonable request.

## Abbreviations

CIS: Clinical Information Systems
EMMS: Electronic Medication Management System
EMR: Electronic Medical Record
FG: Focus group
IS: Information system
ICU: Intensive care unit
IT: Information technology
PhD: Doctor of Philosophy
UTAUT: Unified Theory of Acceptance and Use of Technology
TA: Thematic analysis
TPB: Theory of planned behaviour
TRA: Theory of reasoned action
WOW: Workstations on wheels
