# Supplementary material for: Implementation of an Electronic Medication Management System in a large tertiary hospital: a case of qualitative inquiry
Source: BMC Med Inform Decis Mak. 2021 Jul 27;21:226. doi: 10.1186/s12911-021-01584-w (PMC8314474; doi:10.1186/s12911-021-01584-w)
Supplement: Supplementary file 1 — Additional file 1. Focus group guide. Questions that were used with during FGs. [file 12911_2021_1584_MOESM1_ESM.docx]

**Focus Group Guide**

1. [Constructs: Performance Expectancy (Perceived Usefulness) & Effort Expectancy (Perceived Ease of Use) of the System]: We would like to discuss your opinion about the eMED systems so far.
2. In what ways do you see using the EMMS system enhances your job performance?
3. In what ways do you see using the EMMS system negatively affects your job performance
4. Can you give us some examples of realised (or perceived) benefits of using EMMS?
5. Can you also give us some examples of negative impacts (or cost) of using EMMS to your work?
6. In general, what about the EMMS system that you find it difficult to understand and use?
7. [Constructs: Perceived Benefits & Barriers of Changes]: We would like to discuss the changes to medication management due to the implementation of the EMMS
8. Are there any changes in the way you work as a result of the implementation of EMMS. What are they?
9. What do you consider as the benefits of changing from the old way to the new way of working with EMMS?
10. What do you consider as the barriers of changing from the old way to the new way of working with EMMS?
11. Can you give us some positive examples of the changes to your work and practices in the first few weeks?
12. Can you give us some negative examples of the changes to your work and practices in the first few weeks? Any workarounds you have come up with?
13. [Constructs: Facilitating Condition]: We would like to discuss the environment and factors that may have influenced the acceptance or implementation of EMMS:
14. Organisational Support: What support has been made available to you? Comment on each type of support
15. Organisational Support: What type of support you would like and not being provided so far?
16. Self-Efficacy: How confident are you in your ability to adapt to the new way of working with EMMS?
17. Self-Efficacy: What kind of changes (personally) you think are important to improve your confidence in using EMMS.
18. Compatibility: To what extent the new way of working with EMMS is compatible with the old way of working without EMMS?
19. Compatibility: To what extent the new way of working with EMMS is compatible with your “preferred” way of working with EMMS?
20. Compatibility (Task Technology Fit): What sort of “adjustments” can be made to improve compatibility between EMMS and your preferred way to work.
21. [Constructs: Social Influence]: Social factors that may have affected the attitude and the use of EMMS:
22. Who are the colleagues that are important and influential to your work?
23. What do they think about the new way of working using EMMS?
24. In what way do their opinions change your opinions about the new way of working using EMMS?
25. Who are the people you work with that have a direct effect on your work tasks?
26. Amongst the people who have a direct effect on your work tasks, do they influence your views on EMMS? In what way did they influence you?
