# Supplementary material for: Implementation of an Electronic Medication Management System in a large tertiary hospital: a case of qualitative inquiry
Source: BMC Med Inform Decis Mak. 2021 Jul 27;21:226. doi: 10.1186/s12911-021-01584-w (PMC8314474; doi:10.1186/s12911-021-01584-w)
Supplement: Supplementary file 2 — Additional file 2. Themes, Subthemes and Quotes. Material collated from the analysis of FG data. [file 12911_2021_1584_MOESM2_ESM.docx]

Appendix 2: Themes, Subthemes and Quotes

| **Theme** | **Subtheme** | **Quote** |
| --- | --- | --- |
| Implementation Strategy | Support during the implementation  Training  User engagement/ownership/involvement  Administrative support  Infrastructure  Super-users  Communication among the support staff  Capacity building | “I find the helpline really useful as well. I have called them like five times, I think. They are really helpful.” (P7)  “More practice (with the system) and hands on (training) is needed.” (D4)  “They (Clinicians) were heavily involved in that build process. There was an endocrinologist to put his hands up to help. He was heavily involved, and some of the nurses were involved as well. They accepted the system even prior to our go-live.” (IT1)  “I think the big thing is that we had that governance that supported us. We had that leadership from the executive level from the beginning.” (IT1)  “It has helped a lot, improved what you said that, you know, with giving the medication, the durations, how many days weaning off, its good in a way, I think it’s just a lack of the equipment.” (N2)  “The super users would ensure receiving staff were able to do what they need to do and had someone who is available to answer those sorts of things.” (IT4)  “That instant messaging, can’t speak enough [about it]. Not that I’ve used that much but that’s what lots of other people did (during the roll-out).” (IT2)  “The super user team would ensure that the receiving staff were able to do what they need to do.” (IT4) |
| Organisational Outcome of EMMS | Legibility & Information completeness  Alerts and Prompts  Access to the system  Visibility of information    Workflow | “It’s useful because we can now read their orders a lot clear, we can read them easily.” (N2)  “During the after hour shifts, we have to re-chart lot of meds, that is saving us five to ten minutes per chart. I have found my after-hours shifts much, much more manageable as a result.” (D4)  “Emergency Department has made a folder of the most commonly used medications list (in EMMS), and you can choose from there. Each department is trying to make its own list to save time while ordering medications.” (D2)  “I think it does help. I know it is quite useful to be anywhere in the hospital, I mean if you are in ED and you would have to come all the way up to level six or seven, it is a big deal. So, it really helps in that sense.” (D2)  “It (EMMS)changed the particular way that previously we look at a medication chart I would look at which dose from the pharmacokinetic and pharmacological point of view, which patient is considered lower priority which earlier the higher priority. So I do it high priority.” (P2)  “It changed the particular way that previously we look at a medication chart enabled to assess the clinical context.” (P2) |
| Individual impact of EMMS | Change in the way of working  Accountability  Self-efficacy | “Before I would leave that job. Now do that job straight away, because it’s very easy thing to change.” (D4)  “That is right, you know whom to contact if there is an issue with a dose whereas before when you ring and say “I never charted that”, but (now) at least you can read on the top and see who charted it.” (P3)  “There’s a lot of things each and every one of us has figured out. Like I figured out some things I’m dying to tell it to XX.” (P4) |
| IT product | Design and Build process (Process Design)  Design issues (System Design)  Technical Issues  Workarounds | “So we looked at what the other sites had done and made it better basically.” (IT7)  “That’s the only thing that’s a bit confusing if you don’t read the full order sentences you wouldn’t know if it is paracetamol or paracetamol with codeine.” (N2)  “Our other barrier is mainly to do with listings of product do not match for what we’ve got and having to do all these unnecessary steps of having to change the products.” (P3)  “I think I just like re-charted it like in a slightly different way. It’s a bit time consuming.” (D4) |
| Organisation Culture | Organisational readiness  Communication with colleagues  Cultural factor | “I think the facility has the right culture, and this is only something this hospital just brings itself to make changes to be innovative, develop and work around the new system and develop strategies.” (IT1)  “The interactions with other colleagues like doctors or pharmacist are better than before because of EMMS.” (N1)  “Perhaps we could have some IT person to teach some of our different generation of practitioner how to use the short cut key instead of grabbing the mouse and waste a lot of time.” (P2) |
